# Supplementary material for: Quality Control—A Stepchild in Quantitative Proteomics: A Case Study for the Human CSF Proteome
Source: Biomolecules. 2023 Mar 7;13(3):491. doi: 10.3390/biom13030491 (PMC10046854; doi:10.3390/biom13030491)
Supplement: Supplementary file 1 [file biomolecules-13-00491-s001.zip › Supplement_18.01.2023.pdf]

Supplemental materials

Quality control – a stepchild in quantitative proteomics: a case study for the human CSF proteome

Svitlana Rozanova [1,2]*, Julian Uszkoreit [1,2], Karin Schork[1,2], Bettina Serschnitzki [1,2], Martin Eisenacher[1,2], Lars Tönges[3] and Katalin Barkovits-Boeddinghaus[1,2,&] and Katrin Marcus [1,2,&]

[1] Medical Proteome Analysis, Center for Protein Diagnostics (ProDi), Ruhr University Bochum, 44801 Bochum, Germany
[2] Medizinisches Proteom-Center, Medical Faculty, Ruhr University Bochum, 44801 Bochum, Germany
[3] Neurologische Klinik der Ruhr-Universität Bochum, St. Josef-Hospital, 44791 Bochum, Germany

* Correspondence: svitlana.rozanova@ruhr-uni-bochum.de; Tel.: +49 (0)234 32-18087
[&] Equal contribution

Table of contents

MaCProQC – Mass spectrometry proteomics quality control workflow

Table S1. Comparison of CSF sample preparation protocols used for generation of dataset with qualitative differences.

Table S2. Raw data features taken for the PCA analysis and the scores.

Tables S3. Modifications found using MASCOT error tolerance search.

Table S4. The number of peptides and PGs quantified in the four datasets.

Table S5. Summary of the results obtained for different datasets

Figure S1. Peptide concentration determination using amino acid analysis.

Figure S2. TIC chromatographic profiles for the technical replicates for different CSF datasets.

Figure S3. Raw data quality assessment: quantile analysis of MS1- and MS2- ion intensities.

Figure S4. Box-plots based on the protein intensities.

Figure S5. Analysis of MA-Plots based on raw and LOESS-normalized intensities of PGs.

MaCProQC – Mass spectrometry proteomics quality control workflow

The workflow for the calculation and assembly of quality control (QC) metrics described in the manuscript was implemented as a KNIME workflow. While at the institute a server-based version was used, we published a locally runnable version at https://hub.knime.com/julianu/spaces/MaCProQC/latest/.

The workflow in a nutshell accepts Thermo RAW or mzML files, extracts several QC metrics on these raw data, performs a spectrum identification (using Mascot by Matrixscience) and furthermore uses OpenMS for feature detection and identification mapping. For visualization we created bar plots or scatter plots of PCA (principal component analyses), as shown in the main manuscript. Alternatively, also the pure number in a table could be inspected.

The following metrics, which are also described in [1], were calculated on the unprocessed "raw" file level:

- retention time (RT) duration of the MS run
- number of total MS1 and MS2 events / spectra
- accumulated TICs
- the interval when the first/second/third/fourth 25% of TIC accumulates divided by RT-duration
- the interval for the first/second/third/fourth 25% of all MS respectively MS/MS events divided by RT-duration
- log ratio for 50%ile of TIC changes over 25%ile of TIC changes
- log ratio for 75%ile of TIC changes over 50%ile of TIC changes
- log ratio for largest TIC change over 75%ile of TIC changes
- log ratio for 50%ile of TIC over 25%ile of TIC
- log ratio for 75%ile of TIC over 50%ile of TIC
- log ratio for largest TIC over 75%ile TIC
- maximum of the MS and MS/MS frequency (per minute)
- the 25%ile of MS respectively MS/MS scan peak counts?
- the 50%ile of MS respectively MS/MS scan peak counts?
- the 75%ile of MS respectively MS/MS scan peak counts?
- the fraction of 1, 2, 3, 4, 5 or more charged precursors

On the identifications level, the peptide spectrum matches were filtered on a 1% FDR level using the target-decoy approach on a concatenated 1-to-1 target decoy database using PIA [2] and the following metrics were returned:

- fraction of PSMs with charge 1, 2, 3, 4, 5
- fraction of PSMs with missed cleavage sites for 0, 1, 2, 3 missed cleavages
- total number of (FDR filtered) PSMs, peptides and protein groups

Furthermore, peptide features were detected using the FeatureFinderMultiplex by OpenMS [3] for each separate file and mapped the 1% FDR filtered PSMs from the prior step to these. On this very rudimentary single-file quantitative date the following metrics were extracted:

- the total number of peptide features
- the number of identified peptide features
- the fraction of features with charge 2, 3, 4 and 5 (only these charge states were detected)

[1] Wang X, Chambers MC, Vega-Montoto LJ, Bunk DM, Stein SE, Tabb DL. QC metrics from CPTAC raw LC-MS/MS data interpreted through multivariate statistics. Anal Chem. 2014 Mar 4;86(5):2497-509. doi: 10.1021/ac4034455. Epub 2014 Feb 17. PMID: 24494671; PMCID: PMC3982976.

[2] Uszkoreit J, Maerkens A, Perez-Riverol Y, Meyer HE, Marcus K, Stephan C, Kohlbacher O, Eisenacher M. PIA: An Intuitive Protein Inference Engine with a Web-Based User Interface. J Proteome Res. 2015 Jul 2;14(7):2988-97. doi: 10.1021/acs.jproteome.5b00121. Epub 2015 Jun 10. PMID: 25938255.

[3] Pfeuffer J, Sachsenberg T, Alka O, Walzer M, Fillbrunn A, Nilse L, Schilling O, Reinert K, Kohlbacher O. OpenMS - A platform for reproducible analysis of mass spectrometry data. J Biotechnol. 2017 Nov 10;261:142-148. doi: 10.1016/j.jbiotec.2017.05.016. Epub 2017 May 27. PMID: 28559010.

Table S1. Comparison of CSF sample preparation protocols used for generation of dataset with qualitative differences.

**Dataset**	**CSF, µg of protein**	**Buffer**	**Reduction**	**Alkylation**	**Application on a filter**	**Enzyme, ratio to protein**	**Digestion**	**Stop of the reaction**
Standard in-solution	25	1:1 v/v 0.2% RapiGest (Waters)	5mM DDT 30 min at 60°C	15 mM IAA 30 min at RT in darkness	-	Trypsin (Serva) 1:50	Overnight 37°C	0.5% TFA 45 min 37°C. Centrifugation at 14000xg
Rapid in-solution	25	1:3 v/v 0.2% RapiGest Buffer (Promega)	2 mM TCEP 45 min at 37°C	5 mM IAA 60 min at RT in darkness	-	Trypsin (Promega) 1:10	60 min 70°C	0.5% TFA
Standard FASP	25	1:4 v/v 8M Urea	10 mM DTT 60 min at 37°C	15 mM IAA 30 min at RT in darkness	Vivacon filter (0.5 ml, 10.000 MWCO Sartorius), centrifugation for 15 min at 12000 x g. Washing twice with 200 µl of 8 M Urea and 3 times with 200 µl of Ambic. Re-suspend with 20 µl of 50 mM Ambic.	Trypsin (Serva) 1:50	Overnight 37°C	Elution by adding 50 µl of 50 mM Ambic and 15 min centrifugation at 14000 xg at 18°C. Digestion was stopped by 0.5% TFA.0.5% TFA
Rapid FASP	25	1:3 v/v with 0.2% RapiGest Buffer (Promega)	2 mM TCEP 45 min at 37°C	5 mM IAA 60 min at RT in darkness	Vivacon filter (0.5 ml, 10.000 MWCO Sartorius), centrifugation for 15 min at 12000 x g. Washing twice with 200 µl of Rapid Digest. Re-suspend in 20 µl of Rapid Digest Buffer.	Trypsin (Promega) 1:10	60 min 70°C	Elution by adding 50 µl of 50 mM Ambic and 15 min centrifugation at 14000 xg at 18°C. Digestion was stopped by 0.5% TFA.0.5% TFA

Tables S2. Raw data features taken for the PCA and their influence on the lengths of rotation vectors for PC1 and PC2. A – PCA on raw data, B – PCA on all data.

A

RowID	PC1	PC2	length
total_nr_MS2	0.246	0.047	0.250
RT-MS2-Q3	0.243	-0.089	0.258
MS2-Density-Q1	0.239	0.001	0.239
RT-MS2-Q2	0.233	0.113	0.259
MS2-Density-Q2	0.229	-0.011	0.230
MS2-PrecZ-4	0.229	0.040	0.232
MS1-TIC-Q2	0.226	-0.130	0.261
MS2-Density-Q3	0.205	-0.063	0.214
MS2-PrecZ-5	0.193	-0.076	0.207
RT-TIC-Q3	0.187	0.182	0.261
MS2-PrecZ-more	0.178	-0.073	0.192
MS2-Freq-Max	0.159	0.201	0.256
MS1-TIC-Change-Q2	0.151	-0.125	0.196
accumulated_MS2_TIC	0.150	-0.166	0.223
RT-MS1-Q3	0.135	0.180	0.225
MS2-PrecZ-3	0.126	0.192	0.230
RT-TIC-Q2	0.119	-0.086	0.147
RT-MS1-Q4	0.092	-0.305	0.318
MS1-Density-Q1	0.048	0.306	0.310
RT-TIC-Q1	0.039	0.099	0.107
MS1-Density-Q2	0.037	0.301	0.304
RT-MS2-Q1	0.028	-0.320	0.321
MS2-PrecZ-1	0.000	0.000	0.000
MS1-TIC-Change-Q4	-0.005	0.182	0.182
MS1-TIC-Change-Q3	-0.037	0.087	0.095
RT-MS1-Q2	-0.043	0.318	0.321
RT_duration	-0.064	-0.080	0.102
MS1-Density-Q3	-0.065	0.107	0.126
MS1-Freq-Max	-0.066	0.029	0.072
MS1-TIC-Q4	-0.066	0.238	0.247
accumulated_MS1_TIC	-0.088	-0.265	0.280
RT-MS2-Q4	-0.194	0.202	0.280
MS1-TIC-Q3	-0.219	0.074	0.231
RT-TIC-Q4	-0.223	-0.126	0.256
total_nr_MS1	-0.233	-0.096	0.252
MS2-PrecZ-2	-0.240	-0.043	0.244
RT-MS1-Q1	-0.243	-0.009	0.243

B

RowID	PC1	PC2	length
psmZ-2	0.196	-0.003	0.196
MS2-PrecZ-2	0.186	-0.057	0.194
FeatureZ-2	0.185	-0.064	0.196
psm-missed-0	0.184	0.019	0.185
RT-MS1-Q1	0.180	-0.034	0.183
MS1-TIC-Q3	0.178	0.055	0.186
total_nr_MS1	0.167	-0.118	0.204
RT-TIC-Q4	0.156	-0.144	0.212
RT-MS2-Q4	0.149	0.167	0.224
MS1-Density-Q3	0.064	0.095	0.114
MS1-TIC-Q4	0.061	0.223	0.232
accumulated_MS1_TIC	0.058	-0.255	0.261
RT_duration	0.048	-0.075	0.089
RT-MS1-Q2	0.044	0.294	0.297
MS1-Freq-Max	0.042	0.011	0.044
MS1-TIC-Change-Q3	0.038	0.086	0.093
MS2-PrecZ-1	0.000	0.000	0.000
FeatureZ-1	0.000	0.000	0.000
psmZ-1	0.000	0.000	0.000
MS1-TIC-Change-Q4	-0.007	0.164	0.164
MS1-Density-Q2	-0.020	0.285	0.286
RT-TIC-Q1	-0.025	0.105	0.108
MS1-Density-Q1	-0.026	0.294	0.296
RT-MS2-Q1	-0.029	-0.295	0.296
RT-TIC-Q2	-0.085	-0.072	0.111
FeatureZ-3	-0.085	0.194	0.212
RT-MS1-Q3	-0.085	0.193	0.211
RT-MS1-Q4	-0.086	-0.283	0.296
MS2-PrecZ-3	-0.092	0.201	0.221
MS1-TIC-Change-Q2	-0.103	-0.091	0.138
MS2-Freq-Max	-0.106	0.208	0.234
accumulated_MS2_TIC	-0.113	-0.137	0.178
psm-missed-3	-0.115	-0.103	0.154
RT-TIC-Q3	-0.131	0.190	0.231
number-filtered-psms	-0.134	-0.127	0.184
number-filtered-protein-groups	-0.137	0.021	0.138
MS2-PrecZ-more	-0.141	-0.063	0.154
MS2-Density-Q3	-0.152	-0.038	0.157
FeatureZ-5	-0.154	-0.057	0.164
MS2-PrecZ-5	-0.156	-0.065	0.169
number-filtered-peptides	-0.161	-0.043	0.167
identified_nr_features	-0.162	-0.043	0.168
MS2-Density-Q2	-0.170	0.011	0.170
RT-MS2-Q2	-0.171	0.132	0.216
psmZ-5	-0.173	-0.087	0.194
MS2-PrecZ-4	-0.174	0.052	0.181
psm-missed-2	-0.174	-0.031	0.177
FeatureZ-4	-0.176	0.066	0.188
MS2-Density-Q1	-0.179	0.022	0.180
total_nr_MS2	-0.180	0.072	0.194
MS1-TIC-Q2	-0.182	-0.104	0.210
RT-MS2-Q3	-0.183	-0.059	0.192
psm-missed-1	-0.184	-0.013	0.185
psmZ-4	-0.185	-0.060	0.194
psmZ-3	-0.194	0.042	0.199

Tables S3. Modifications found using MASCOT error tolerance search

A. Standard in-solution

			Std in-sol 1	Std in-sol 2	Std in-sol 3	Std in-sol 4	Std in-sol 5
Modification	Delta	Site	Total matches	Total matches	Total matches	Total matches	Total matches
Carbamidomethyl	57.021464	C	1558	1666	1568	1589	1617
Non-specific cleavage		-	798	806	848	863	801
Carbamidomethyl	57.021457	N-term	498	528	543	548	493
Oxidation	15.994915	M	428	483	467	487	448
Gly	57.021464	K	289	288	262	284	259
Gly	57.021464	S	253	270	245	268	268
Deamidated	0.984016	N	252	266	243	258	254
Gly	57.021464	T	192	197	218	220	186
Carbamidomethyl	57.021464	E	132	128	147	145	115
GlyGly	114.04293	C	131	127	130	132	124
Dethiomethyl	-48.00337	M	112	124	114	102	120
Gln->pyro-Glu	-17.02653	N-term	78	89	86	89	84
Oxidation	15.994915	P	63	89	72	58	61
Carboxymethyl	58.005479	C	66	78	66	80	71
Carbamidomethyl	57.021464	Y	66	68	70	74	68
Carboxymethyl	58.005481	N-term	62	73	62	65	55
Carbamidomethyl	57.021464	D	56	68	66	79	59
GlyGly	114.04293	K	51	62	57	56	57
Carbamidomethyl	57.021464	H	42	54	56	59	56

B. Rapid Digest in-solution

			Rapid in-sol 1	Rapid in-sol 2	Rapid in-sol 3	Rapid in-sol 4	Rapid in-sol 5
Modification	Delta	Site	Total matches	Total matches	Total matches	Total matches	Total matches
Carbamidomethyl	57.02146	C	2290	2175	2396	2208	2388
Oxidation	15.99492	M	727	647	747	704	729
Non-specific cleavage		-	583	559	586	583	563
Carbamidomethyl	57.02146	N-term	329	368	321	320	305
Gly	57.02146	S	213	226	198	197	211
Gly	57.02146	T	197	208	210	187	179
Deamidated	0.984016	N	187	174	186	172	177
Gly	57.02146	K	170	196	178	172	163
Carbamidomethyl	57.02146	E	122	123	128	94	112
GlyGly	114.0429	C	102	114	94	91	102
Oxidation	15.99492	P	101	87	106	104	98
Gln->pyro-Glu	-17.0265	N-term	81	81	82	90	86
Cys->Dha	-33.9877	C	64	57	73	61	60
Carboxymethyl	58.00548	C	46	52	70	62	59
Dethiomethyl	-48.0034	M	58	61	50	52	49

C. Standard FASP

			Std FASP 1	Std FASP 2	Std FASP 3	Std FASP 4	Std FASP 5
Modification	Delta	Site	Total matches	Total matches	Total matches	Total matches	Total matches
Carbamidomethyl	57.02146	C	1905	2185	2621	2030	2327
Oxidation	15.99492	M	936	930	1087	935	1061
Non-specific cleavage		-	672	641	773	744	747
Carbamyl	43.00582	N-term	763	423	128	190	507
Gly	57.02146	S	171	165	142	184	158
Carbamidomethyl	57.02146	N-term	131	143	106	155	150
Gly	57.02146	T	127	141	123	125	128
Deamidated	0.984016	N	130	121	131	106	117
Oxidation	15.99492	P	70	102	116	81	107
Gln->pyro-Glu	-17.0265	N-term	82	89	111	94	99
Carbamidomethyl	57.02146	E	97	82	58	77	78
Carbamyl	43.00581	K	114	77	26	29	86
Gly	57.02146	K	56	62	58	65	54

D. Rapid Digest FASP

			Rapid FASP1	Rapid FASP2	Rapid FASP3	Rapid FASP4	Rapid FASP5
Modification	Delta	Site	Total matches	Total matches	Total matches	Total matches	Total matches
Carbamidomethyl	57.02146	C	2043	2012	1934	2114	2078
Non-specific cleavage		-	866	1022	1066	873	986
Oxidation	15.99492	M	582	633	707	584	612
Gly	57.02146	S	180	157	153	168	170
Deamidated	0.984016	N	154	201	234	143	165
Gln->pyro-Glu	-17.0265	N-term	74	86	100	86	80
Oxidation	15.99492	P	69	80	131	62	160
Carbamidomethyl	57.02146	N-term	69	60	61	64	61
Gly	57.02146	K	51	43	39	48	43
Gly	57.02146	T	50	41	49	47	37

Table S4. The number of peptides and PGs quantified in the four datasets.

Protocol	Quantified peptides	Peptides overlap for the 5 replicates	Quantified PGs	PGs overlap for the 5 replicates
Standard in-solution	3200	1825 (57%)	515	332 (64%)
Rapid in-solution	3902	2275 (58%)	539	369 (68%)
Standard FASP	4678	2299 (49%)	615	386 (63%)
Rapid FASP	3080	1493 (48%)	496	277 (56%)

Table S5. Summary of the results obtained for different datasets.

Level of assessment	Parameter	Standard in-solution	Rapid in-solution	Standard FASP	Rapid FASP
Raw data (MaCProQC tool)	Number of MS1 scans	9347<br>CV=2.1%	9218<br>CV=0.7%	9273<br>CV=1.8%	10363<br>CV=6.7%
	Number of MS2 scans	38121<br>CV=1.9%	39294<br>CV=0.8%	38902<br>CV=1.2%	35438<br>CV=5.9%
	MS1 accumulated intensity	6.06E+12<br>CV=13.3%	7.86E+12<br>CV=19.0%	8.51E+12<br>CV=17.4%	1.01E+13<br>CV=13.2%
	TIC quantile distribution	One outlier (shift of the 1st and 2nd quantiles)	No severe difference for the replicates	No severe difference for the replicates	Chromatogram intensity variation for all 5 replicates (shift of the 3rd and 4th quantiles)
	Precursor charge state distribution	Stable precursor charge distribution for the replicates.	Stable precursor charge distribution for the replicates. Enhanced number of highly charged ions.	Highly varying precursor charge distribution for the replicates.	Slight variations in precursor charge distribution for the replicates.
	Summary PCA	Low intragroup variability.	Low intragroup variability.	Low intragroup variability.	High intragroup variability.
Identification level (MaCProQC tool)	Number of PSMs	6851<br>CV=2.3%	8619<br>CV=3.3%	9592<br>CV=6.7%	7302<br>CV=7.7%
	Number of identified peptides	2526<br>CV=3.2%	3109<br>CV=2.2%	3413<br>CV=6.5%	2366<br>CV=9.2%
	Number of identified PGs	416<br>CV=2.0%	444<br>CV=3.4%	495<br>CV=4.4%	381<br>CV=10.9%
	PSMs with 1-3 missed cleavages	14.6%	22%	19.6%	6.4%
	Distribution of PSMs based on missed cleavages	Stable distribution for the replicates.	Stable distribution for the replicates.	Highly varying distribution for the replicates.	Stable distribution for the replicates.
	Summary PCA	Low intragroup variability.	Low intragroup variability.	Slightly higher intergroup separation	High intragroup variability.
QC for label-free quantification	Number of quantified peptides in total	3200	3902	4678	3080
	Data completeness (number of overlapping peptides)	1825	2275	2299	1493
	Number of quantified protein groups in total	515	539	615	496
	Data completeness (number of overlapping PGs)	332	369	386	277
	Similarity of the replicates based on clustering (Pearson correlation coefficient, peptides)	0.96-0.97	0.96-0.98	0.90 - 0.96	0.88 - 0.97
	Similarity of the replicates based on clustering (Pearson correlation coefficient, PGs)	0.97-0.98	0.96-0.98	0.96-0.98	0.82 - 0.97
	Efficiency of data normalization	Both LOESS and MaxQuant LFQ normalization worked comparably well, improving the replicate clustering.	Both LOESS and MaxQuant LFQ normalization worked comparably well, improving the replicate clustering.	Both LOESS and MaxQuant LFQ normalization worked comparably well, improving the replicate clustering.	Most divergent dataset. Both LOESS and MaxQuant LFQ normalization were not effective.

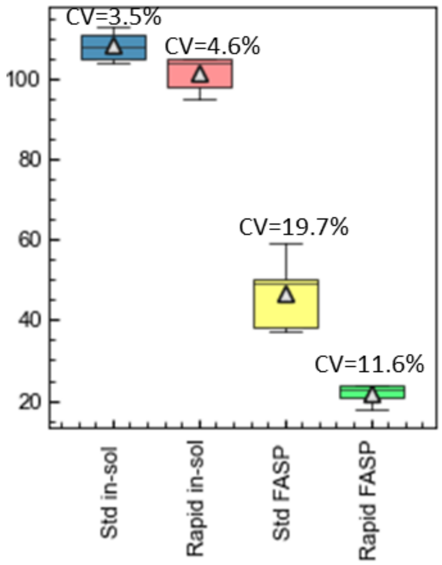

Figure S1. Peptide concentration determination using amino acid analysis.

Recovery of 25µg of CSF protein (based on Bradford assay) after different digestion approaches. The percentage of recovery higher than 100% is attributed to the differences in the protein concentration determination before and after digestion: Bradford assay and AAA respectively. Std in-sol – standard in solution digestion, Rapid in sol – rapid in solution digestion, Std FASP - standard filter added preparation, Rapid FASP - rapid filter added preparation, CV – coefficient of variation.

A. Standard in solution digestion

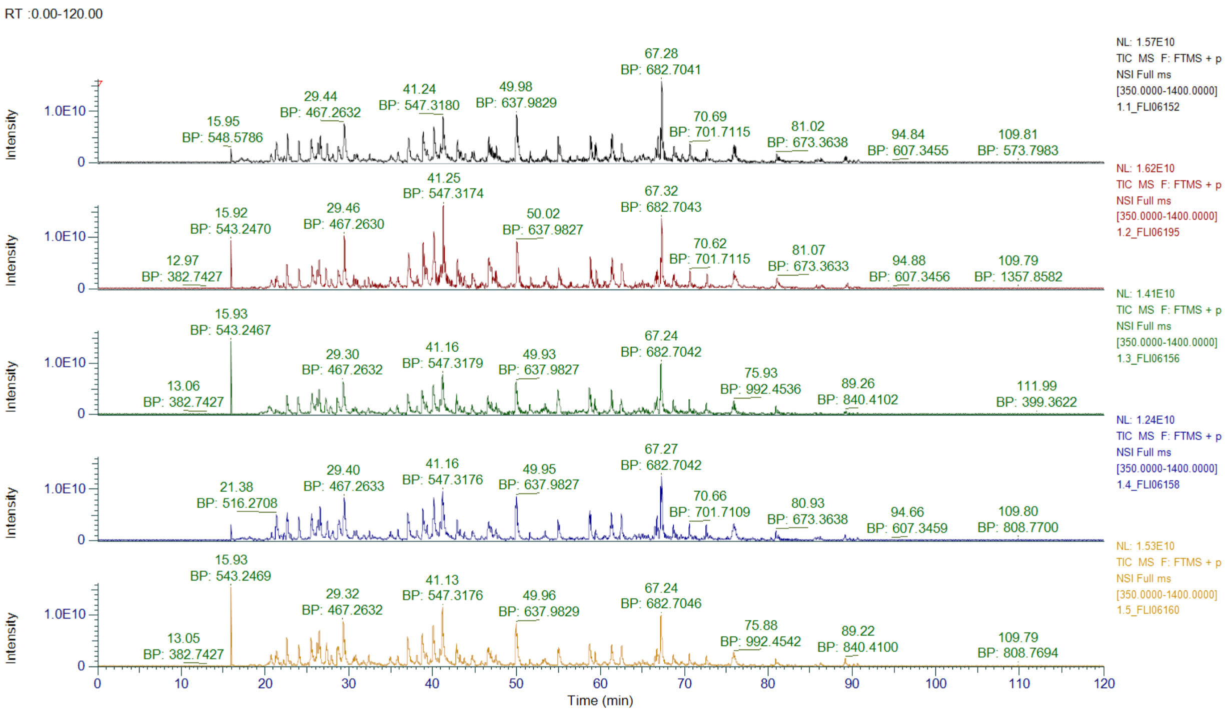

B. Rapid in solution digestion

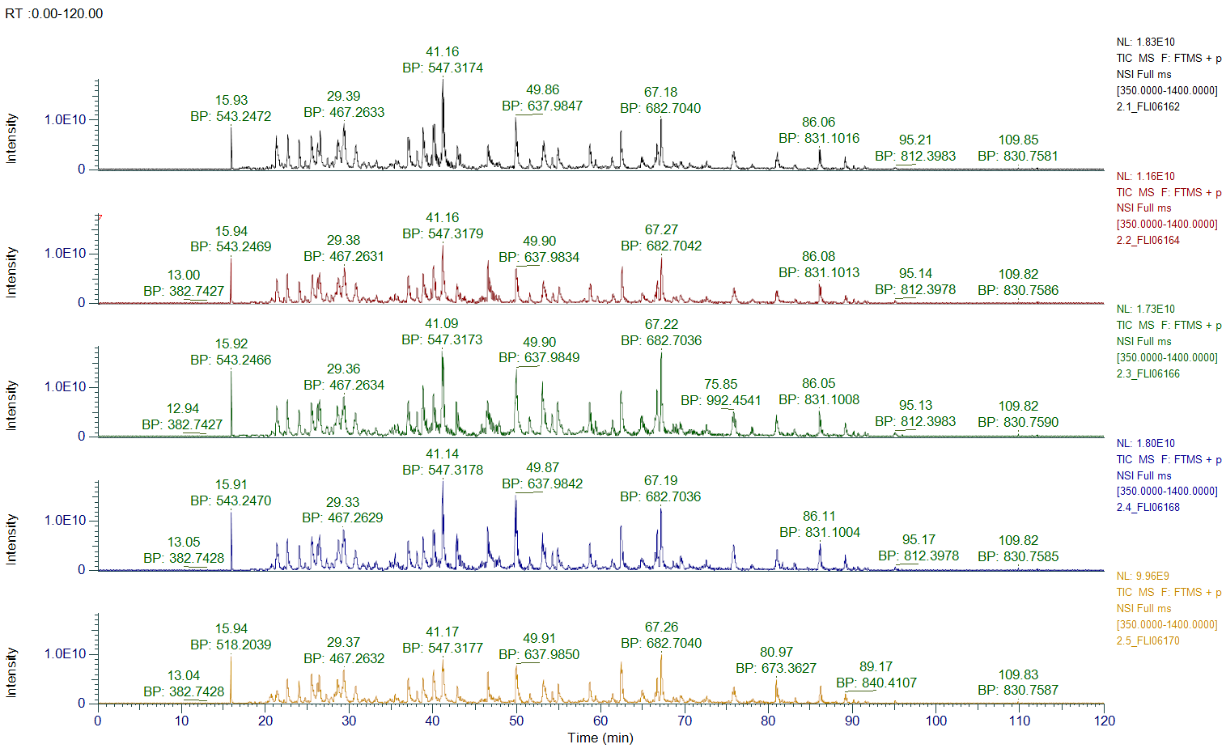

C. Standard FASP

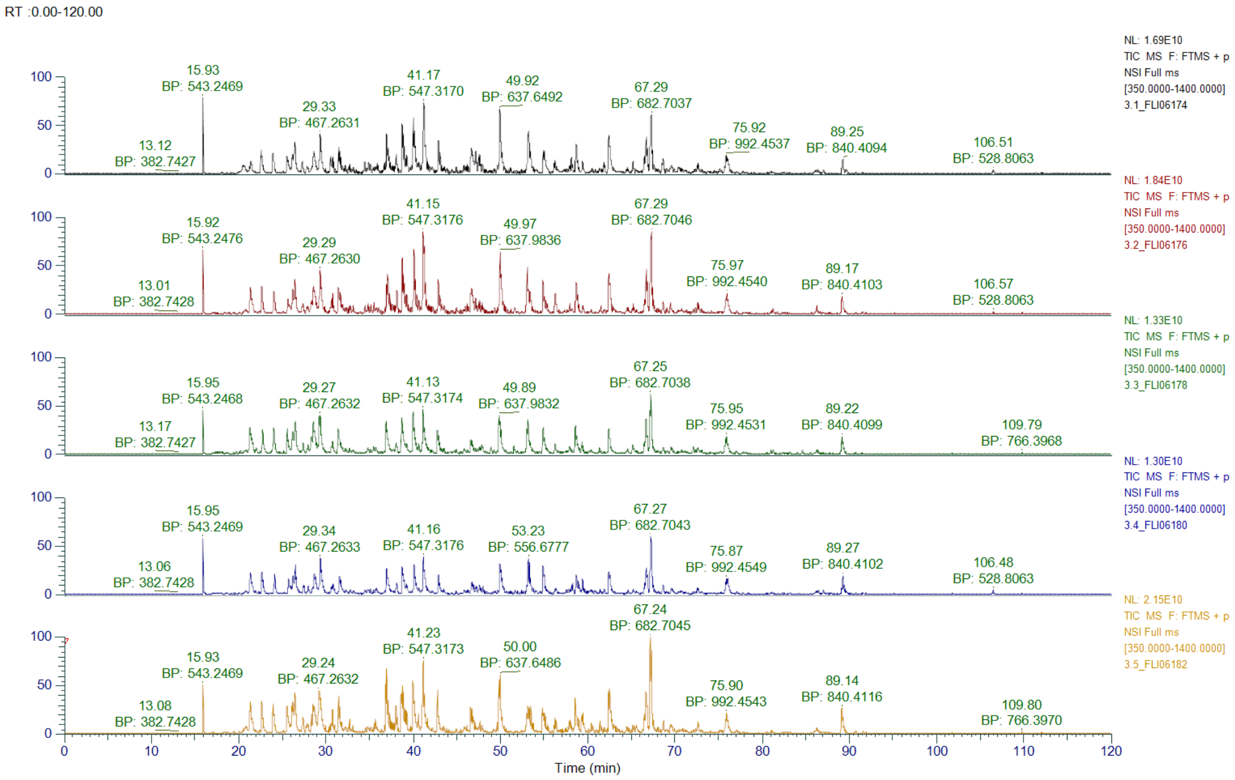

D. Rapid FASP

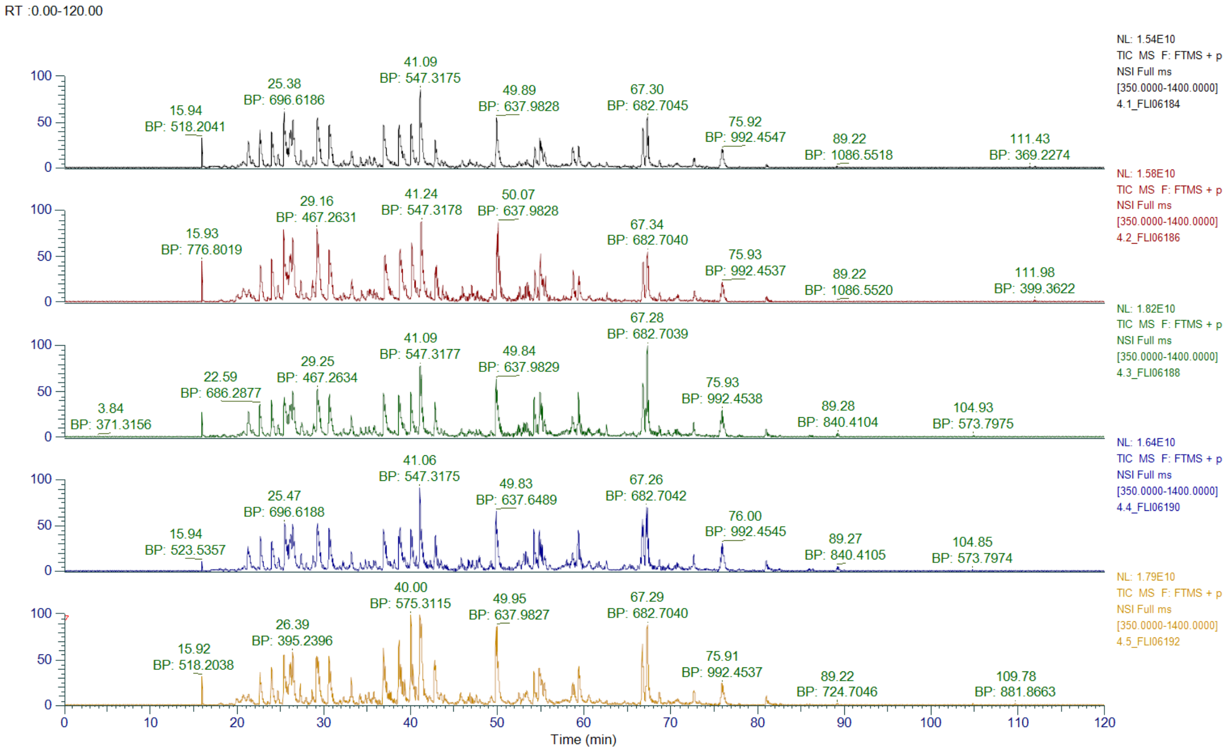

Figure S2. TIC chromatographic profiles for the technical replicates for different CSF datasets: A – standard in solution digestion, B – rapid in solution digestion, C - standard filter added preparation, D - rapid filter added preparation.

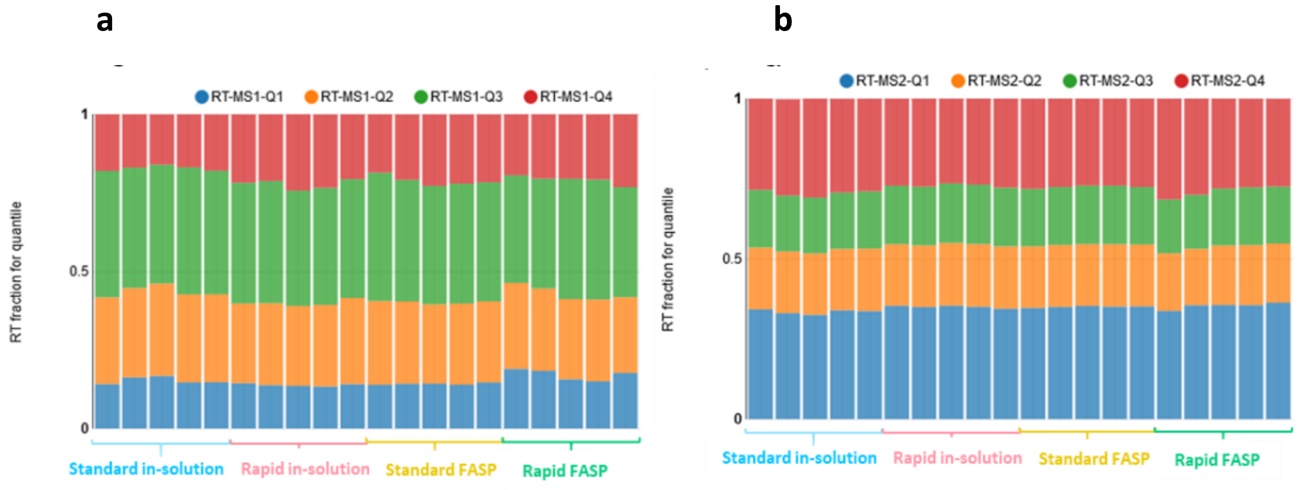

Figure S3. Raw data quality assessment: quantile analysis of MS1- and MS2- ion intensities. The similarity of the obtained chromatograms was assessed in *MaCProQC* using TIC quantile analysis on MS1 (a) and MS2 (b) levels, where the quantiles reflect the fraction of the retention time (RT) referring to the 1st (RT-TIC-Q1), 2nd (RT-TIC-Q2), 3rd (RT-TIC-Q3) and 4th (RT-TIC-Q4) quarter of the total run's intensity. Slight shifts were seen for the rapid FASP on both MS1 and MS2 levels.

A. Non normalyzed intensities

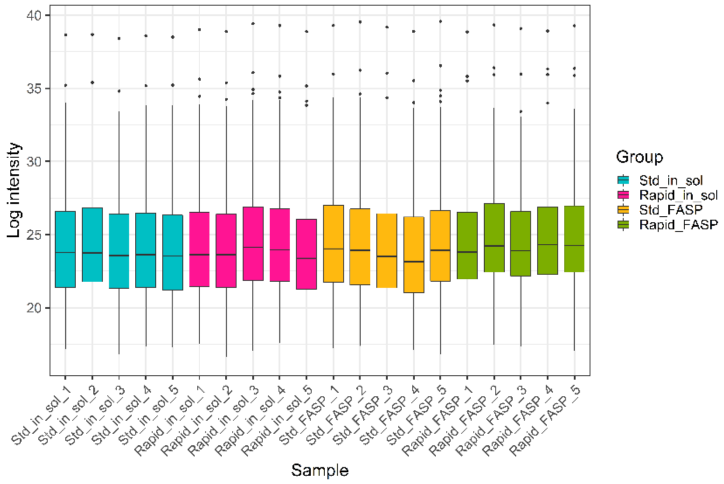

B. LOESS normalyzed intensities

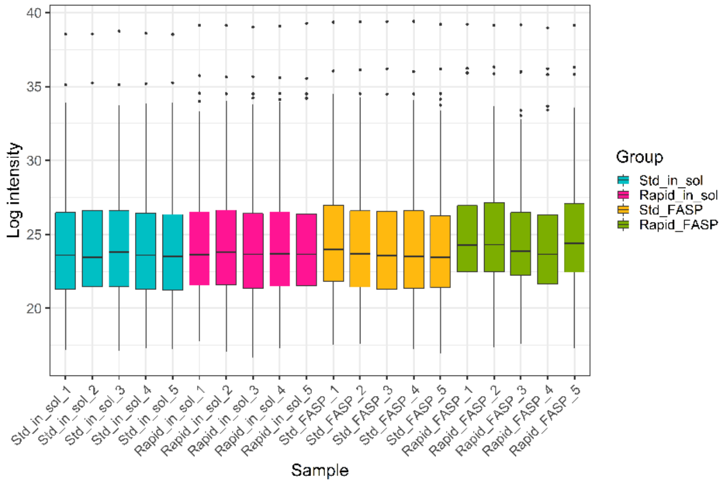

C. Normalyzed LFQ intensities

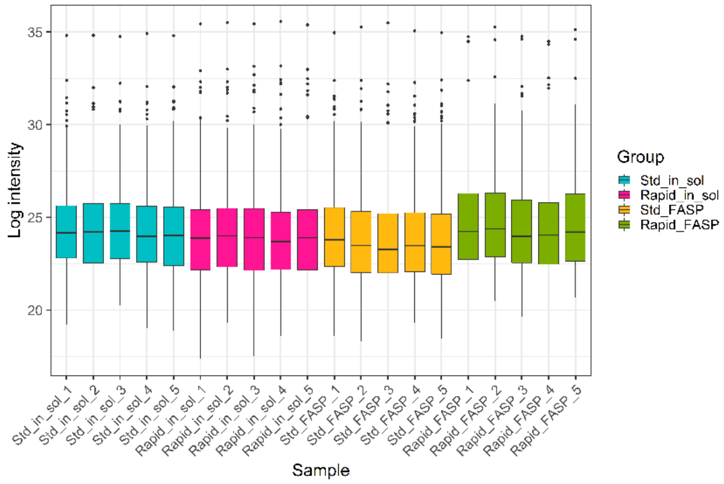

Figure S4. Box-plots based on the protein intensities: A- non-normalized; B – separate LOESS normalization; C - LFQ normalization.

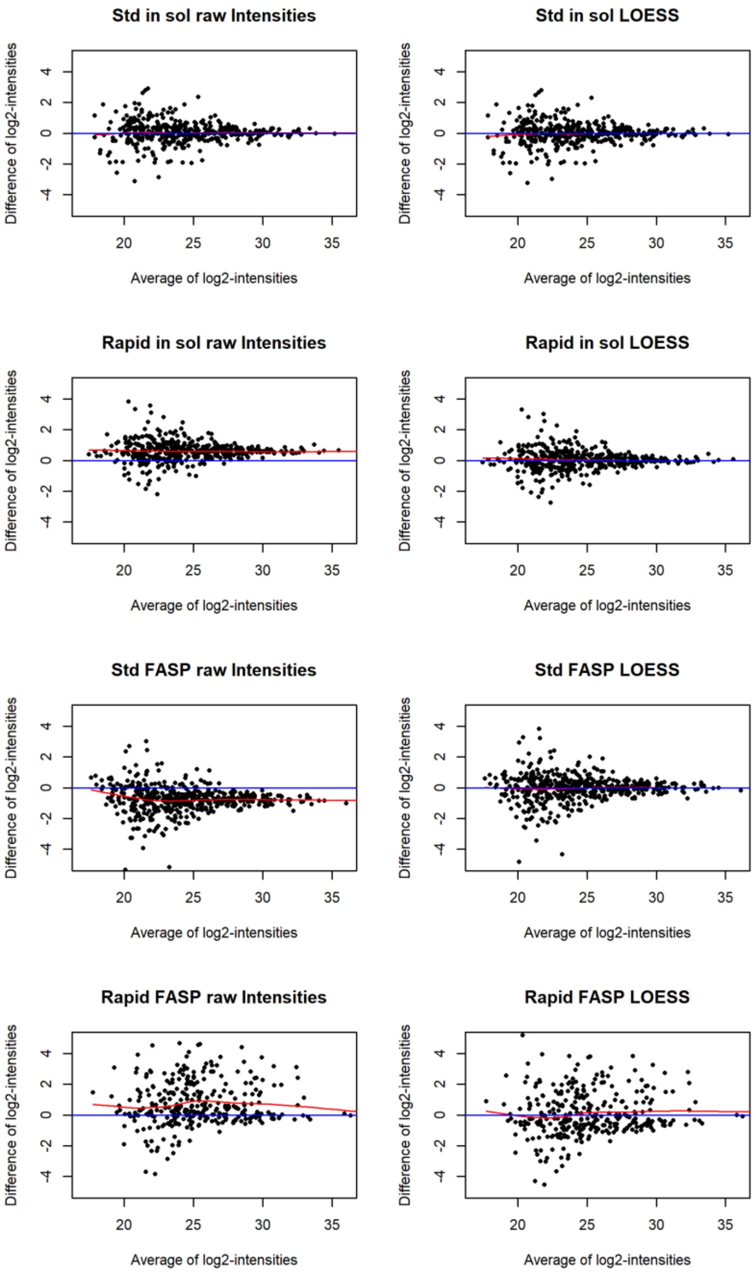

Figure S5. Analysis of MA-Plots based on raw and LOESS-normalized intensities of PGs.

Typical MA-Plots comparing the raw (left) and LOESS-normalized intensities (right) of PGs for the 4th and 5th replicate of the respective dataset. Each point represents one protein (low-abundant proteins on the left and the high-abundant proteins on the right). Proteins with a large difference between the two compared samples are at the top or the bottom, those with a small difference are around y = 0 (blue line). The red line is the local regression line fitted to the point cloud. Ideally, this line would be equal to the blue horizontal line at y = 0. If the red line is shifted from the blue line, this indicates a technical bias. No technical bias was detected for the standard in-solution dataset replicates, while the other dataset had a data bias. For rapid FASP, the spread of the data points along the y-axis is the highest, indicating higher level of variability. Furthermore, the point cloud in the MA-Plot is not as symmetric as for the other groups. LOESS normalization minimized the variance for the standard in-solution and FASP (the red local regression line is closer to the horizontal blue line at y = 0). However, the normalization cannot reduce the spread for Rapid FASP and here still the point cloud is asymmetrical to the blue line.
